# Using Deep Learning Algorithms to Grade Hydronephrosis Severity: Toward a Clinical Adjunct

**DOI:** 10.3389/fped.2020.00001

**Published:** 2020-01-29

**Authors:** Lauren C. Smail, Kiret Dhindsa, Luis H. Braga, Suzanna Becker, Ranil R. Sonnadara

**Affiliations:** ^1^Department of Psychology, Neuroscience & Behaviour, McMaster University, Hamilton, ON, Canada; ^2^Office of Education Science, McMaster University, Hamilton, ON, Canada; ^3^Department of Surgery, McMaster University, Hamilton, ON, Canada; ^4^Research and High Performance Computing, McMaster University, Hamilton, ON, Canada; ^5^Vector Institute for Artificial Intelligence, Toronto, ON, Canada; ^6^Division of Urology, Department of Surgery, McMaster University, Hamilton, ON, Canada; ^7^Division of Urology, Department of Surgery, McMaster Children's Hospital, Hamilton, ON, Canada; ^8^McMaster Pediatric Surgery Research Collaborative, McMaster University, Hamilton, ON, Canada; ^9^Centre for Advanced Research in Experimental and Applied Linguistics, McMaster University, Hamilton, ON, Canada

**Keywords:** hydronephrosis, machine learning, deep learning, ultrasound, diagnostic imaging, grading, diagnostic aid, teaching aid

## Abstract

Grading hydronephrosis severity relies on subjective interpretation of renal ultrasound images. Deep learning is a data-driven algorithmic approach to classifying data, including images, presenting a promising option for grading hydronephrosis. The current study explored the potential of deep convolutional neural networks (CNN), a type of deep learning algorithm, to grade hydronephrosis ultrasound images according to the 5-point Society for Fetal Urology (SFU) classification system, and discusses its potential applications in developing decision and teaching aids for clinical practice. We developed a five-layer CNN to grade 2,420 sagittal hydronephrosis ultrasound images [191 SFU 0 (8%), 407 SFU I (17%), 666 SFU II (28%), 833 SFU III (34%), and 323 SFU IV (13%)], from 673 patients ranging from 0 to 116.29 months old (*M*_age_ = 16.53, *SD* = 17.80). Five-way (all grades) and two-way classification problems [i.e., II vs. III, and low (0–II) vs. high (III–IV)] were explored. The CNN classified 94% (95% CI, 93–95%) of the images correctly or within one grade of the provided label in the five-way classification problem. Fifty-one percent of these images (95% CI, 49–53%) were correctly predicted, with an average weighted F1 score of 0.49 (95% CI, 0.47–0.51). The CNN achieved an average accuracy of 78% (95% CI, 75–82%) with an average weighted F1 of 0.78 (95% CI, 0.74–0.82) when classifying low vs. high grades, and an average accuracy of 71% (95% CI, 68–74%) with an average weighted F1 score of 0.71 (95% CI, 0.68–0.75) when discriminating between grades II vs. III. Our model performs well above chance level, and classifies almost all images either correctly or within one grade of the provided label. We have demonstrated the applicability of a CNN approach to hydronephrosis ultrasound image classification. Further investigation into a deep learning-based clinical adjunct for hydronephrosis is warranted.

## Introduction

Machine learning is a field of research with far reaching applications that is generating considerable interest in medicine (1, 2). Deep learning, a subset of machine learning, is a general term for an algorithm that trains a many layered network to learn hierarchical feature representations from raw data. Due to the hierarchical nature of deep learning models, complex functions can be learned to solve difficult classification problems that were previously unsolvable by classic machine learning algorithms (3). Deep convolutional neural networks (CNNs) are a type of deep learning algorithm that are well-suited to computer vision tasks (3) due to their ability to take advantage of the multi-scale spatial structure of images (4). This makes CNN models an attractive candidate architecture for tackling medical imaging problems. In particular, they offer a promising avenue for creating clinical adjuncts to help train physicians, and flag/grade challenging diagnostic cases.

Prenatal hydronephrosis (HN) is a condition that involves accumulation of urine with consequent dilatation of the collecting system in fetuses. It is the most frequent neonatal urinary tract abnormality, occurring in 1–5% of all newborn babies (5). HN is detected by prenatal ultrasound (US) imaging and can be caused by several underlying conditions, such as uteropelvic junction obstruction or vesico-ureteral reflux (6). Although many cases eventually resolve on their own, in severe forms, afflicted infants may require surgical intervention (7, 8), and failure to intervene can result in loss of renal function (9, 10).

All patients with prenatal HN are normally evaluated after birth by postnatal renal ultrasonography to determine HN severity and the best course of treatment. Appropriate HN grading is important, as misclassification of any patient into the inappropriate HN category can lead to incorrect management and unnecessary testing since treatment is directly dependent on HN severity. Given the need for accurate and unambiguous classification of HN, numerous HN grading systems have been developed (11). However, poor inter-rater reliability (12, 13), particularly for intermediate HN grades, suggests that grading still relies on subjective interpretation of ultrasound images, as clear and objective criteria have not been fully established.

Owing to the ability of deep learning algorithms to classify images into diagnostic categories based solely on data-driven pattern recognition, the main purpose of this study was to extend on our previous work (14) to investigate whether deep learning algorithms can effectively grade the severity of HN using a prospectively collected HN database and separate them into 5 main classes. Secondary investigations were also conducted to assess whether the same model can effectively discriminate between low and high HN grades (SFU 0, I, II vs. III, IV), and between moderate (SFU II vs. III) cases. The results of this study may provide important insights into whether deep learning is a promising avenue of future study for discriminating different grades of HN, and developing clinical adjuncts. Given that our models were trained on images with human expert-generated training labels, we hypothesized that our deep learning model would perform at or very close to that of a human expert at HN grading. This would validate our method as a potential training tool for medical students and as an adjunctive tool for clinical experts.

## Materials and Methods

### Study Population and Exclusion Criteria

Our database consists of 2-dimensional renal B-mode US images from an ongoing large prospective cohort study involving all patients diagnosed with prenatal HN who were referred to a tertiary care pediatric hospital. The database contains one sagittal US image per patient visit, spanning 687 patients. Each image was assigned a grade according to the Society for Fetal Urology (SFU) system, one of the most widely adopted HN classification systems (15), ranging from 0 (normal kidney) to IV (severe HN with parenchymal thinning). Grades were provided by three separate physicians (2 fellowship trained pediatric urologists and 1 fellowship trained pediatric radiologist—agreement *K* = 90%) with discrepancies resolved by consensus. From these 687 patients, 2,492 sagittal renal US images were collected. Seventy-two images from 14 patients were excluded due to poor image quality (e.g., blurry, large annotation overlaid, no visible kidney), leaving 2,420 sagittal US images from 673 patients (*N*_female_ = 159, *N*_male_ = 514) ranging from 0 to 116.29 months old (*M*_age_ = 16.53, *SD* = 17.80) to be included in the analysis. Of these, 191 were labeled as SFU 0, 407 as SFU I, 666 as SFU II, 833 as SFU III, and 323 as SFU IV. Ethics clearance for this study was obtained through the Research Ethics Board.

### Preprocessing

Preprocessing is a crucial step in machine learning, as standardizing images and taking simple steps to reduce noise and non-discriminative variability improves the ability of models to learn relevant information. In this study, all images were cropped to remove any annotations and blank space in the margins. The images were then despeckled using the bi-directional FIR-median hybrid despeckling filter to remove speckle noise from the images (16). Despeckling is a standard preprocessing technique for US images since speckle noise is caused by interference between the US probe and reflecting US waves. Finally, the image pixel values were normalized between 0 and 1, and all images resized to 256 × 256 pixels to provide a consistent image input size into our network. The final image size was chosen based on the smallest dimension of the cropped images to ensure that images were not stretched.

Data augmentation is a common approach to reducing overfitting and improving classification performance for small datasets (3, 17). It works by introducing variations on each image during training so as to build robustness into the model. In this study, we augment the data by rotating each image up to 45°, performing horizontal and vertical flips with a 50% probability, and shifting the image vertically and horizontally up to 20%.

### Model Architecture

A CNN is a type of neural network that has been particularly successful in computer vision applications. CNNs are constructed from alternating convolutional layers and pooling layers. The structure of a CNN is inspired by that of the mammalian visual system, where earlier cortical areas receive input from small regions of the retina and learn simple local features such as edges, while regions at progressively higher levels in the visual system have correspondingly broader receptive fields, and learn complex features such as shape detectors. In a CNN, convolutional layers learn multiple local features of an image by processing it across many overlapping patches, while pooling layers summate the filter responses from the previous layer, thereby compressing the representations learned by the preceding convolutional layer to force the model to filter out unimportant visual information. As in the visual system, successive convolutional layers have progressively larger receptive fields, permitting more complex, and abstract image features to be learned in higher layers of the network. In classification models a standard multilayer perceptron, made up of a few fully connected layers of neurons (called dense layers) receives the learned image representation from the convolutional layers and attempts to classify the image. The entire network is trained using backpropagation, a neural network learning procedure which iteratively updates the strengths of the connections between layers of neurons in order to minimize classification error on the training data. For a detailed explanation of how CNNs work and are designed, see Le Cun et al. (18).

**Figure 1:**
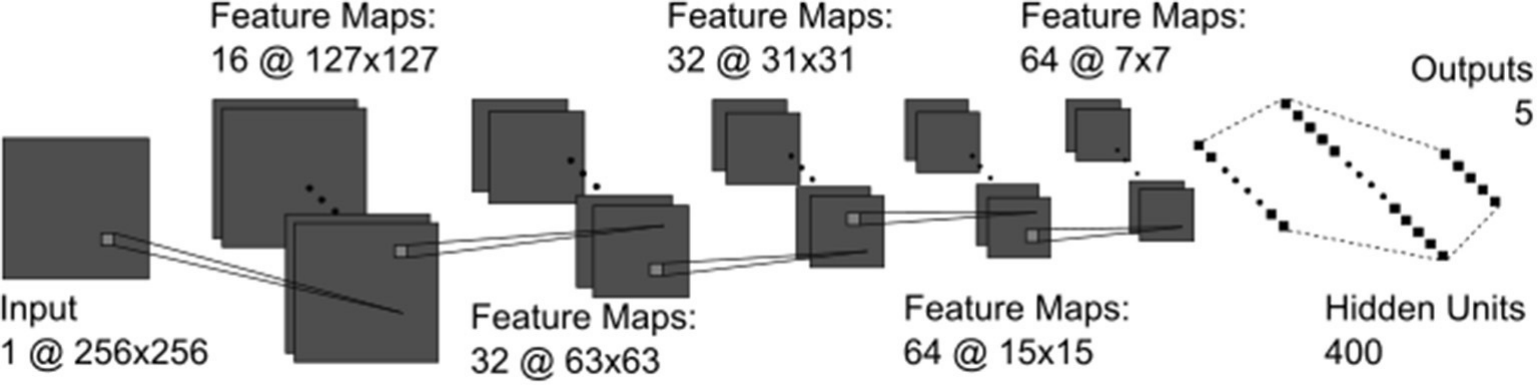
The CNN architecture containing all convolutional (dark gray) and fully connected (black) layers. The convolutional kernels (light gray squares) were 3 × 3 pixels in all layers.

The CNN model used in the current study was developed using the Keras neural network API with Tensorflow (19, 20). The final architecture contained five convolutional layers, a fully connected layer of 400 units, and a final output layer where the number of units was equal to the number of classes for the given task (i.e., five or two) (Figure 1). The architecture was determined by experimenting with five-way SFU HN classification. The output unit/class with the highest overall final activation was used as the model's prediction and was compared against the provided label to assess performance. See Supplementary Materials for a description of all technical details.

### Model Training and Evaluation

Five-way (all SFU grades) and binary classification tests were conducted using 5-fold cross validation. See Supplementary Materials for a description of this process. The binary classification tests were selected due to their clinical relevance and included distinguishing between mild (0, I, and II) and severe (III and IV) HN grades, and between moderate grades (II vs. III). Layer-wise relevant propagation (21) was used to visualize model output.

## Results

**Figure 2:**
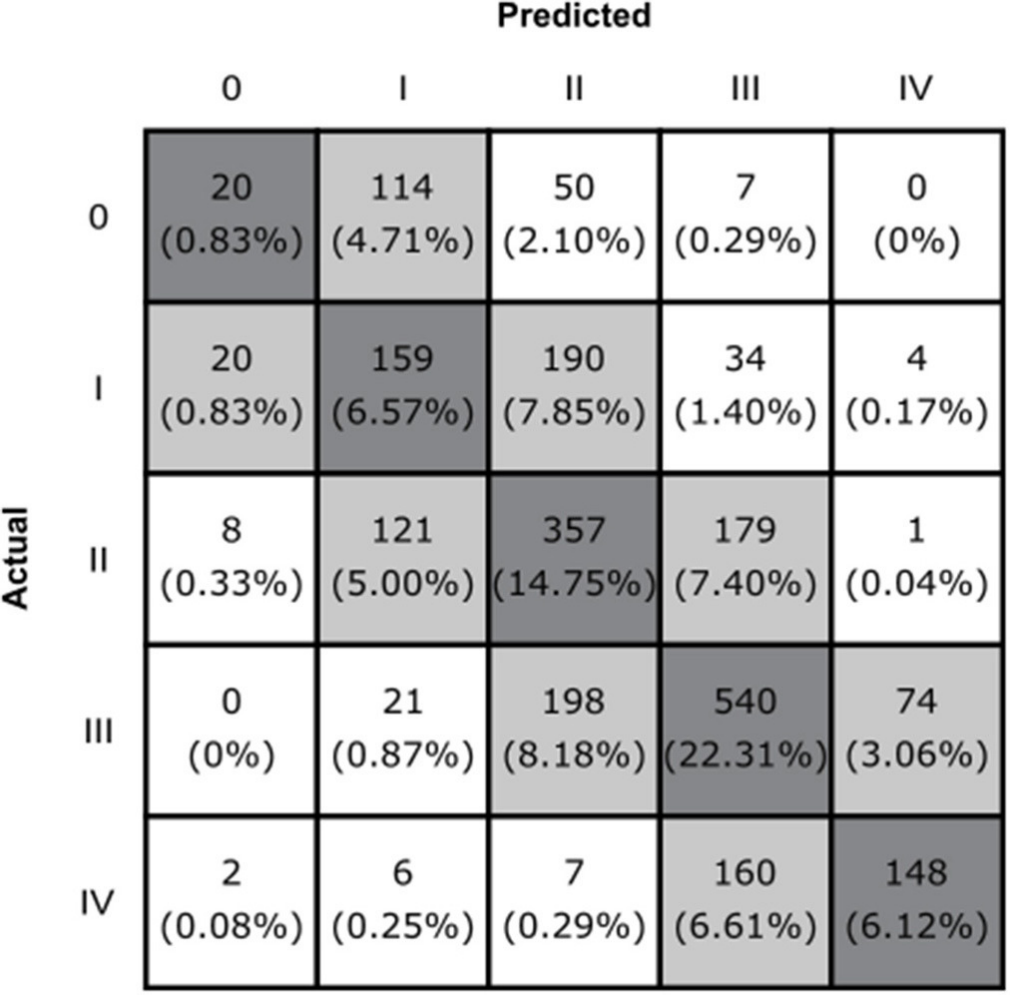
The confusion matrix of the CNN model. Boxes along the diagonal in gray represent the number (percentage) of cases where the CNN made the correct classification decision. Light gray boxes represent the cases where the CNN was incorrect by one grade, and white boxes indicate cases where the CNN was incorrect by two or more grades.

Our model achieved an average five-way classification accuracy of 51% (95% CI, 49–53%), and an average weighted F1 score of 0.49 (95% CI, 0.47–0.51). Furthermore, 94% (95% CI, 93–95%) of images were either correctly classified or within one grade of the provided label (Figure 2).

Our model classified mild vs. severe HN with an average accuracy of 78% (95% CI, 75–82%), and an average weighted F1 of 0.78 (95% CI, 0.74–0.82). When differentiating between moderate grades (SFU II and III), our model achieved an average accuracy of 71% (95% CI, 68–74%) and an average weighted F1 score of 0.71 (95% CI, 0.68–0.75). See Table 1 for a comprehensive overview of model performance.

**Table 1:** CNN model classification results averaged across the 5-folds.

**Classification problem**	**Accuracy (%)**	**Sensitivity**	**Specificity**	**PPV**	**F1**
Five-way (0 to IV)	51 (49–53)				0.49 (0.47–0.51)^a^
SFU 0		0.11 (0–0.21)	0.99 (0.97–1.00)	0.26 (0.05–0.47)	0.15 (0.01–0.29)
SFU 1		0.39 (0.35–0.43)	0.87 (0.84–0.90)	0.39 (0.34–0.44)	0.38 (0.35–0.42)
SFU II		0.54 (0.43–0.65)	0.75 (0.72–0.79)	0.45 (0.42–0.49)	0.48 (0.43–0.53)
SFU III		0.65 (0.60–0.70)	0.76 (0.74–0.78)	0.59 (0.53–0.65)	0.61 (0.56–0.66)
SFU IV		0.46 (0.29–0.62)	0.96 (0.94–0.98)	0.65 (0.54–0.75)	0.52 (0.38–0.66)
Mild (0, I, II) vs. Severe (III, IV)	78 (75–82)				0.78 (0.74–0.82)^a^
Mild		0.89 (0.82–0.96)	0.66 (0.51–0.81)	0.75 (0.69–0.81)	0.81 (0.78–0.84)
Severe		0.66 (0.51–0.81)	0.89 (0.82–0.96)	0.87 (0.80–0.94)	0.73 (0.64–0.82)
SFU II vs. SFU III	71 (68–74)				0.71 (0.68–0.75)^a^
SFU II		0.76 (0.60–0.92)	0.67 (0.52–0.82)	0.67 (0.59–0.75)	0.69 (0.63–0.75)
SFU III		0.67 (0.52–0.82)	0.76 (0.60–0.92)	0.80 (0.73–0.87)	0.71 (0.65–0.77)

*The 95% confidence intervals are given in parentheses*.

a *Weighted average*.

## Discussion

We investigated the potential of deep CNN to create clinical adjuncts for HN. This was achieved by testing our model's ability to classify HN US images. We tested our model's performance on three different classification tasks that are relevant to clinical practice. These results, along with their potential clinical implications, are discussed below.

### Five-Way Classification Performance

Our model achieved an average five-way classification accuracy that was well above chance level (51%). In practice, physicians usually have access to multiple different US images at different angles, as well as patient histories, and are therefore able to grade the US image by combining information from multiple views and timepoints. Although we are unable to compare our model's performance directly to a physician, achieving this level of accuracy with a single US image is very promising.

**Figure 3:**
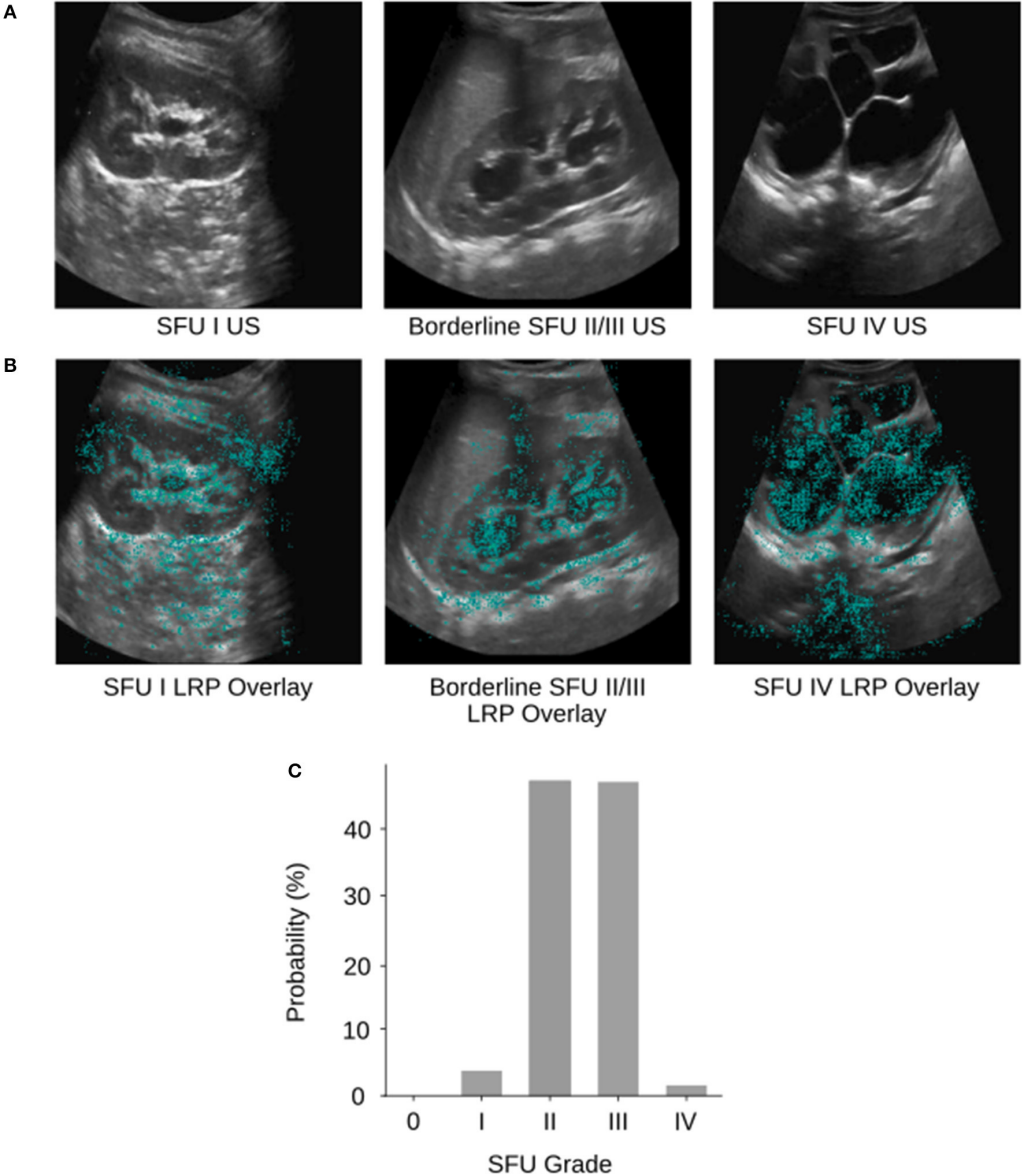
**(A)** Example SFU I, borderline SFU II/III, and SFU IV US images from the database. **(B)** The corresponding layerwise relevance propagations of each of the example images. Layer-wise relevance propagations give a sparse representation of pixel importance. Propagations were visualized as heat maps and overlaid on top of the gray-scale input US images. The cyan colored pixels indicate regions that the CNN heavily relied upon for classification. **(C)** The corresponding softmax output probability distribution of the borderline SFU II/III US image. The image was labeled as SFU grade III by physicians; however, the CNN predicted SFU grade II which was incorrect. We can see based on the probability distribution that the model “thought” SFU grade II and III were almost equally likely but had to select one grade as its prediction. This behavior is analogous to that of physicians and can be partially explained by the poor inter-rater reliability and subjectivity of the SFU system (i.e., intrinsic limitations of that classification).

The model classified 94% (95% CI, 93–95%) of images either correctly or within one grade of the correct/provided label. Further investigation into the output of our model reveals that there are many borderline images where there is not an obvious choice for which class the image belongs to (e.g., Figures 3A,C). In cases such as these where two grades possible are, it must choose a single HN grade according to the SFU system, much like a physician (12, 13).

Considering that HN grading can be challenging, and that subjective assessments are used to differentiate between borderline cases (12, 13), we would argue that solely relying on whether the model's predictions matched the provided SFU labels is an incomplete assessment of our model's performance. Instead, the percentage of cases that are either “correct” of within one grade of the provided label (94%) is a more representative metric of our model's true performance. The nearly block-diagonal structure of the confusion matrix supports this (Figure 2) and indicates that the model is learning useful information for HN classification.

### Binary Classification Performance

Discriminating between moderate HN grades is known to be challenging (12, 13), and therefore we wanted to investigate our model's performance on this same task. When comparing mild (0, I, II) and severe (III, IV) HN images, our model achieved an average accuracy of 78%, which is well above chance level. When the model discriminated between moderate grades (II and III), which is less reliable for physicians (12, 13), performance only dropped to 71%. There is no direct comparison to be made against physician accuracy, however, considering the known difficulties in distinguishing between moderate HN grades (12, 13), these results are encouraging.

### Interpretability

We visualized regions of the HN US images that the CNN found important for five-way classification in a sample of images using layer-wise relevance propagation (21) from the DeepExplain toolbox (22). Layer-wise relevance propagation allows us to determine which features in the image contribute most strongly to the CNNs output (Figure 3B). Cyan pixels indicate that the model heavily relied on those features to classify the image. Visualizing can be used to validate whether our model is learning appropriate features that correspond with the SFU grading system and interpret its inner workings. Interpretability is crucial as we develop deep learning based clinical adjuncts since physicians will need to be able to understand why a model made a decision, rather than just blindly following the algorithm.

Of the examples we tested, we can see that our model is learning features that correspond appropriately with the SFU system (e.g., renal parenchyma, calyces), however, in some cases it is also relying on regions outside of the kidney. This can likely be attributed to image noise, and therefore removing the noise with segmentation (i.e., finding regions of interest in the image) would ensure that the model is only relying on appropriate regions for classification. However, the model may be finding relevant features outside of those from the SFU classification system that are clinically relevant but not normally considered, and so this finding warrants further investigation.

### Implications for Clinical Practice

Machine learning and deep learning models have been successfully applied in the context of HN to predict the need for surgical intervention (1), and the necessity of diuretic nuclear renography (2). More broadly, machine learning and deep learning have been used in the field of pediatric urology to classify between different kidney diseases (23), and between diseased and normal kidneys (24). In addition, deep learning has recently been used to perform automatic kidney segmentation in ultrasound imaging (25). Due to the different problems being evaluated in each of the studies, a direct comparison in performance cannot be made. It is important to highlight that along with investigating different questions, and therefore having differing levels of chance performance (i.e., 50 vs. 20% in the current study), these studies also differ from the current study in that many of these papers are asking objective questions (e.g., Was surgery required?) and are therefore able to utilize objective labels in their models. As discussed previously, the lack of objective ground truth in the current study presents challenges in interpreting the true performance of our model, and likely contributes to our model's lower accuracy metric as compared to other papers.

Considering the issue of subjectivity, our model's current level of performance in classifying HN is promising and in line with previous research from our group (14). Our findings suggest that applying these algorithms into clinical practice through decision aids and teaching aids has potential. It is important to clarify that we anticipate that deep learning models like the one presented here will 1 day be used to support physicians rather than replace them, as human-level reliability and generalizability remains a major challenge for medical applications (26). We outline below two new ways that we expect deep learning models can be applied to benefit clinical practice in the future.

#### Decision Aids

In clinical practice, decision aids are used to assess the structure of interest, and then provide its estimate of disease probability. Physicians are then free to use this estimate as they wish. To our knowledge, patient management is always left up to the physician, and the aids act more like a second opinion. Studies have shown that the combined synergistic effects of the decision aid and physician knowledge greatly improved the diagnostic accuracy (27). In the context of HN, we expect that the second opinion from the decision aid would be particularly useful for borderline cases, since currently consensus decisions are required to resolve these cases.

#### Teaching Aids

Deep learning models can also be used to develop teaching aids for trainees to teach and provide them with feedback on how to grade HN US images. These teaching features can be created by exploiting the rich information that these algorithms contain. For example, a deep learning-based teaching aid could provide trainees with informative feedback based on the inner workings of the algorithm to tell trainees whether their diagnosis was correct. Furthermore, the teaching aid could highlight parts of the image with a heat-map using visualization methods, such as layer-wise relevance propagation, to indicate which regions were of clinical importance, and to what degree. A teaching aid would alleviate at least some of the need for direct physician feedback and would allow trainees to work through examples at their own pace to maximize learning.

### Limitations and Future Work

Considering that the current dataset was small by deep learning standards, slightly imbalanced, and only contained one image per patient visit, our model still achieved moderate to good accuracy across the different classification problems. This suggests that a richer and larger dataset could lead to even better performance and an eventual deep learning based clinical adjunct for HN. Future work should also investigate HN classification at the patient level and consider the time series in the data. HN patients are followed across time, and the trends in their HN severity provide physicians with important information that is incorporated into their clinical decision making. We would expect that providing a deep learning model with time series data would benefit model performance as well. Additionally, a model could convey its level of uncertainty in its diagnosis, flagging to the physician that this image merited a closer examination or additional measurements.

We applied relatively little preprocessing to our images, therefore future studies should investigate whether segmentation, a commonly recommended preprocessing technique, reduces model noise and improves performance (25). Within the current classification model, layer-wise relevance propagation revealed that regions outside of the kidney were contributing to model output. Further investigation on the impact of segmentation whereby the model is constrained to extract features from the kidney that correspond with the SFU grading system should elucidate whether these findings are attributable to image noise or useful features.

## Conclusions

The purpose of the current study was to explore whether deep learning can effectively classify HN US images and separate them into 5 main categories. Overall, our model performs well above chance level across all classifications, categorizing images either correctly, or within one grade of the provided label. The model was also capable of discriminating well between mild and severe grades of HN, which has important clinical implications. The results of the current study suggest that CNNs can be applied to grade HN US images effectively, and that further investigation into using deep learning to grade HN US images is warranted. With further model refinement, and by addressing the limitations of our current data set, we expect that our model can be used to develop effective clinical adjuncts to improve clinical practice.

## Data Availability Statement

The datasets generated for this study are available on request to the corresponding author.

## Ethics Statement

The studies involving human participants were reviewed and approved by Hamilton Integrated Research Ethics Board. Written informed consent to participate in this study was provided by the participants' legal guardian/next of kin.

## Author Contributions

LS was responsible for data analysis and writing the first draft of the manuscript. LB provided clinical oversight for the project, and was responsible for acquisition and curation of the dataset used for model training. All authors were responsible for the design of the study, interpretation of the data, and writing the final manuscript.

### Conflict of Interest

The authors declare that the research was conducted in the absence of any commercial or financial relationships that could be construed as a potential conflict of interest.

## References

[1] Lorenzo AJ Rickard M Braga LH Guo Y Oliveria JP. Predictive analytics and modeling employing machine learning technology: the next step in data sharing, analysis and individualized counseling explored with a large, prospective prenatal hydronephrosis database. Urology. (2018) 123:204–9. 10.1016/j.urology.2018.05.041 29964127

[2] Cerrolaza JJ Peters CA Martin AD Myers E Safdar N Linguraru MG. Quantitative ultrasound for measuring obstructive severity in children with hydronephrosis. J Urol. (2016) 195:1093–9. 10.1016/j.juro.2015.10.173 26551298

[3] Krizhevsky A Sutskever I Hinton GE ImageNet classification with deep convolutional neural networks. In: Pereira F Burges CJC Bottou L Weinberger KQ editors. Advances in Neural Information Processing Systems 25. Lake Tahoe, NV: Curran Associates Inc (2012). p. 1097–105.

[4] Le Cun Y Bottou L Bengio Y Haffner P Gradient-based learning applied to document recognition. Proc IEEE. (1998) 86:2278–324. 10.1109/5.726791

[5] Woodward M Frank D. Postnatal management of antenatal hydronephrosis. BJU Int. (2002) 89:149–56. 10.1046/j.1464-4096.2001.woodward.2578.x 11849184

[6] Montini G Tullus K Hewitt I. Febrile urinary tract infections in children. N Engl J Med. (2011) 365:239–50. 10.1056/NEJMra1007755 21774712

[7] Yang Y Hou Y Niu ZB Wang CL. Long-term follow-up and management of prenatally detected, isolated hydronephrosis. J Pediatr Surg. (2010) 45:1701–6. 10.1016/j.jpedsurg.2010.03.030 20713223

[8] Braga LH McGrath M Farrokhyar F Jegatheeswaran K Lorenzo AJ. Associations of initial society for fetal urology grades and urinary tract dilatation risk groups with clinical outcomes in patients with isolated prenatal hydronephrosis. J Urol. (2017) 197:831–7. 10.1016/j.juro.2016.08.099 27590478

[9] González R Schimke CM. The prenatal diagnosis of hydronephrosis, when and why to operate? Arch Esp Urol. (1998) 51:575–9. 9773587

[10] Hanna MK. Antenatal hydronephrosis and ureteropelvic junction obstruction: the case for early intervention. Urology. (2000) 55:612–5. 10.1016/S0090-4295(00)00460-X 10792063

[11] Nguyen HT Benson CB Bromley B Campbell JB Chow J Coleman B. Multidisciplinary consensus on the classification of prenatal and postnatal urinary tract dilation (UTD classification system). J Pediatr Urol. (2014) 10:982–98. 10.1016/j.jpurol.2014.10.002 25435247

[12] Rickard M Easterbrook B Kim S Farrokhyar F Stein N Arora S. Six of one, half a dozen of the other: a measure of multidisciplinary inter/intra-rater reliability of the society for fetal urology and urinary tract dilation grading systems for hydronephrosis. J Pediatr Urol. (2017) 13:80.e1–5. 10.1016/j.jpurol.2016.09.005 27916387

[13] Keays MA Guerra LA Mihill J Raju G Al-Asheeri N Geier P. Reliability assessment of society for fetal urology ultrasound grading system for hydronephrosis. J Urol. (2008) 180:1680–3. 10.1016/j.juro.2008.03.107 18708207

[14] Dhindsa K Smail LC McGrath M Braga LH Becker S Sonnadara RR Grading prenatal hydronephrosis from ultrasound imaging using deep convolutional neural networks. In: 15th Conference on Computer and Robot Vision. Toronto, ON: IEEExplore (2018). p. 80–7. Available online at: https://bibbase.org/network/publication/dhindsa-smail-mcgrath-braga-becker-sonnadara-gradingprenatalhydronephrosisfromultrasoundimagingusingdeepconvolutionalneuralnetworks-2018 (accessed July 12, 2018).

[15] Nguyen HT Herndon CDA Cooper C Gatti J Kirsch A Kokorowski P. The society for fetal urology consensus statement on the evaluation and management of antenatal hydronephrosis. J Pediatr Urol. (2010) 6:212–31. 10.1016/j.jpurol.2010.02.205 20399145

[16] Nieminen A Heinonen P Neuvo Y. A new class of detail-preserving filters for image processing. IEEE Trans Pattern Anal Mach Intell. (1987) 9:74–90. 10.1109/TPAMI.1987.4767873 21869378

[17] Simard PY Steinkraus D Platt JC Best practices in convolutional neural networks applied to visual document analysis. In: Seventh International Conference on Document Analysis and Recognition, 2003 (Edinburgh) (2003). p. 958–62. 10.1109/ICDAR.2003.1227801

[18] Le Cun Y Bengio Y Hinton G Deep learning. Nature. (2015) 521:436–44. 10.1038/nature14539 26017442

[19] Chollet F Keras (2015). Available online at: http://keras.io (accessed September 20, 2018).

[20] Abadi M Agarwal A Barham P Brevdo E Chen Z Citro C TensorFlow: Large-Scale Machine Learning on Heterogeneous Distributed Systems (2016). Available online at: http://arxiv.org/abs/1603.04467 (accessed March 14, 2019).

[21] Bach S Binder A Montavon G Klauschen F Müller KR Samek W. On pixel-wise explanations for non-linear classifier decisions by layer-wise relevance propagation. PLoS ONE. (2015) 10:e0130140. 10.1371/journal.pone.0130140 26161953 PMC4498753

[22] Ancona M Ceolini E Öztireli C Gross M Towards Better Understanding of Gradient-Based Attribution Methods for Deep Neural Networks (2017). Available online at: http://arxiv.org/abs/1711.06104 (accessed September 20, 2018).

[23] Yin S Peng Q Li H Zhang Z You X Liu H. Multi-instance deep learning with graph convolutional neural networks for diagnosis of kidney diseases using ultrasound imaging. In: Greenspan H Tanno R Erdt M Arbel T Baumgartner C Dalca A editors. Uncertainty for Safe Utilization of Machine Learning in Medical Imaging and Clinical Image-Based Procedures. Cham: Springer International Publishing (2019). p. 146–54. 10.1007/978-3-030-32689-0_15 PMC6938161 31893285

[24] Zheng Q Furth SL Tasian GE Fan Y. Computer-aided diagnosis of congenital abnormalities of the kidney and urinary tract in children based on ultrasound imaging data by integrating texture image features and deep transfer learning image features. J Pediatr Urol. (2019) 15:75.e1–7. 10.1016/j.jpurol.2018.10.020 30473474 PMC6410741

[25] Sivanesan U Braga LH Sonnadara RR Dhindsa K Unsupervised Medical Image Segmentation with Adversarial Networks: From Edge Diagrams to Segmentation Maps (2019). Available online at: http://arxiv.org/abs/1911.05140 (accessed December 03, 2019).

[26] Dhindsa K Bhandari M Sonnadara RR. What's holding up the big data revolution in healthcare? BMJ. (2018) 363:k5357. 10.1136/bmj.k5357 30593447

[27] Doi K. Computer-aided diagnosis in medical imaging: historical review, current status and future potential. Comput Med Imaging Graph. (2007) 31:198–211. 10.1016/j.compmedimag.2007.02.002 17349778 PMC1955762

