# Supplementary material for: Using Deep Learning Algorithms to Grade Hydronephrosis Severity: Toward a Clinical Adjunct
Source: Front Pediatr. 2020 Jan 29;8:1. doi: 10.3389/fped.2020.00001 (PMC7000524; doi:10.3389/fped.2020.00001)
Supplement: Supplementary file 1 [file Data_Sheet_1.docx]

Supplementary Material

# Supplementary Methods Details

## CNN Architecture

The CNN contained five convolutional layers, a fully connected layer of 400 units, and a final output layer where the number of units was equal to the number of classes for the given task (i.e. five or two). Global average pooling was applied after the final convolutional layer to decrease the number of parameters and to speed up learning. (1) Each layer of the model used a rectifying nonlinearity (ReLU) activation function to speed up the training time, (2) except for the final layer which used a Softmax activation function to output the final model prediction. The output unit/class with the highest overall final activation was used as the model’s prediction and was compared against the provided label to assess performance.

A learning rate of 0.01 and a decay value of 10^-5^ was used to train the model to minimize the ordinal categorical cross entropy using the nonlinear optimization algorithm Adam. (3) Three hundred epochs were used to train the model to convergence. The first convolutional layer contained 16 filters, the second and third layers contained 32 filters and the fourth and fifth layers contained 64 filters (Figure 1). Each filter in the entire model was 3x3 pixels in size and had a stride length of 1 pixel. Batch normalization and max pooling were applied after each convolutional layer to make training more robust and efficient, (4) and reduce the size of the image for subsequent layers respectively. (5) We used batch sizes of 25 images, and max pooling inputs patches of 3x3 pixels with a stride length of 2x2 pixels. Finally, a dropout of 0.5 was used for the fully connected layer to reduce overfitting and promote the learning of independent features. (6)

## Cross-validation

Each model was trained and evaluated using a 5-fold cross-validation loop. Cross-validation is a technique whereby a model is repeatedly trained and tested on different sets of the data to evaluate how generalizable its results are. In the current study, the sagittal images were shuffled and then split into five sets, with all images belonging to a patient remaining in the same set to ensure that within-patient similarities would not cause the model to overfit. Each of the five sets was used as a test set once. Model performance was assessed using classification accuracy, sensitivity, specificity, positive predictive value (PPV), and F1 score averaged across the five folds. The F1 score is the weighted average of precision and sensitivity, which provides a better assessment of the model’s performance when there is an imbalance in the number of samples of each class. (7) For a five-way classification problem, an F1-score of 0.2 corresponds to chance accuracy. No statistical comparisons were made against physician performance since no gold standard measure exists.

# References

1. Lin M, Chen Q, Yan S. Network In Network. arXiv:13124400 [cs] [Internet]. 2013 Dec 16 [cited 2018 Jul 18]; Available from: http://arxiv.org/abs/1312.4400

2. Goodfellow I, Bengio Y, Courville A. Deep Learning [Internet]. MIT Press; 2016. Available from: http://www.deeplearningbook.org

3. Kingma DP, Ba J. Adam: A Method for Stochastic Optimization. arXiv:14126980 [cs] [Internet]. 2014 Dec 22 [cited 2018 Jul 11]; Available from: http://arxiv.org/abs/1412.6980

4. Ioffe S, Szegedy C. Batch Normalization: Accelerating Deep Network Training by Reducing Internal Covariate Shift. arXiv:150203167 [cs] [Internet]. 2015 Feb 10 [cited 2018 May 12]; Available from: http://arxiv.org/abs/1502.03167

5. Nagi J, Ducatelle F, Caro GAD, Cireşan D, Meier U, Giusti A, et al. Max-pooling convolutional neural networks for vision-based hand gesture recognition. In: 2011 IEEE International Conference on Signal and Image Processing Applications (ICSIPA). 2011. p. 342–7.

6. Srivastava N, Hinton G, Krizhevsky A, Sutskever I, Salakhutdinov R. Dropout: A Simple Way to Prevent Neural Networks from Overfitting. J Mach Learn Res. 2014 Jan;15(1):1929–1958.

7. Davis J, Goadrich M. The Relationship Between Precision-Recall and ROC Curves. In: Proceedings of the 23rd International Conference on Machine Learning [Internet]. New York, NY, USA: ACM; 2006 [cited 2018 May 13]. p. 233–240. (ICML ’06). Available from: http://doi.acm.org/10.1145/1143844.1143874
